# Research progress of novel bio-denitrification technology in deep wastewater treatment

**DOI:** 10.3389/fmicb.2023.1284369

**Published:** 2023-10-04

**Authors:** Shan Huang, Yuling Fu, Huimin Zhang, Chuqiao Wang, Chenglong Zou, Xiuguo Lu

**Affiliations:** School of Civil Engineering and Architecture, East China Jiao Tong University, Nanchang, China

**Keywords:** novel bio-denitrification technology, deep wastewater denitrification, coupling process, denitrification performance, low-carbon

## Abstract

Excessive nitrogen emissions are a major contributor to water pollution, posing a threat not only to the environment but also to human health. Therefore, achieving deep denitrification of wastewater is of significant importance. Traditional biological denitrification methods have some drawbacks, including long processing times, substantial land requirements, high energy consumption, and high investment and operational costs. In contrast, the novel bio-denitrification technology reduces the traditional processing time and lowers operational and maintenance costs while improving denitrification efficiency. This technology falls within the category of environmentally friendly, low-energy deep denitrification methods. This paper introduces several innovative bio-denitrification technologies and their combinations, conducts a comparative analysis of their denitrification efficiency across various wastewater types, and concludes by outlining the future prospects for the development of these novel bio-denitrification technologies.

## 1. Introduction

With the ongoing advancements in industrialization and urbanization, excessive nitrogen emissions from industrial, domestic, and agricultural wastewater have resulted in environmental issues like eutrophication, unpleasant odors, and deterioration of water quality in surface bodies (Qu et al., 2019; Ceulemans et al., 2023; Xia and Yan, 2023). The primary denitrification methods encompass physicochemical and biological approaches. The physicochemical method mainly involves ion exchange, adsorption, chemical precipitation, and redox reactions, often requiring the addition of adsorbents, catalysts, and ion exchangers to achieve nitrogen removal. However, adsorption is sensitive to water quality variations, and adsorbents have a limited lifespan. Ion exchangers can lead to secondary pollution, while the use of catalysts increases treatment expenses (Soldatov et al., 2007; Tarpeh et al., 2017).

In contrast, the bio-denitrification process has gained popularity due to its cost-effectiveness, minimal by-product generation, dependable operation, and environmental compatibility (Zhang et al., 2016). Traditional bio-denitrification technology effectively treats nitrogen-containing wastewater through bio-nitrification (Eq. 1) and bio-denitrification (Eq. 2) processes. However, it necessitates aeration and the addition of organic carbon sources, which not only increase costs but can also contribute to secondary pollution, contradicting the low-carbon paradigm (Sanjrani et al., 2022). Consequently, the research and application of innovative bio-denitrification technologies hold significant importance for environmental preservation, ecological equilibrium, wastewater reclamation, energy conservation, and addressing emerging environmental challenges. This paper will introduce several novel bio-denitrification technologies and their integration processes, assess their denitrification performance, and summarize their research advancements in the field of wastewater denitrification.


(1) $${NH}_{4}^{+}+2O_{2}\to 2H^{+}+H_{2}O+{\text{NO}}_{3}^{-}$$

(2) $$2{\text{NO}}_{3}^{-}+10H\left(\text{electron}\;\text{donor}\right)\to 2{OH}^{-}+4H_{2}O+N_{2}$$

## 2. Novel bio-denitrification process

### 2.1. Novel bio-denitrification technology

#### 2.1.1. Short-cut nitrification denitrification

Voets et al. (1975) made a pivotal discovery regarding the accumulation of nitrite (NO_2_^−^-N) during the nitrification process and introduced the concept of short-cut nitrification denitrification (SCND) technology. The fundamental principle involves the conversion of ammonia nitrogen (NH_4_^+^-N) into NO_2_^−^-N through controlled reaction parameters (Eq. 1), followed by direct reduction of NO_2_^−^-N to nitrogen (N_2_) through denitrification (Eq. 2; Huang, 2021). This innovation results in significant savings, reducing aeration requirements by 25% and organic carbon source needs by 40% when compared to traditional bio-denitrification processes (Kornaros et al., 2010). The key to SCND technology lies in regulating the nitrification process to stop at the nitrite stage.

It has been demonstrated that the control of reaction temperature, pH, dissolved oxygen (DO) concentration, and sludge age can effectively modulate the growth conditions of ammonia-oxidizing bacteria (AOB) and nitrite-oxidizing bacteria (NOB), thereby facilitating NO_2_^−^-N accumulation (Zhu et al., 2008). Currently, SCND technology has found practical applications in NH_4_^+^-N removal from landfill leachate (Zhang et al., 2020), sludge digestion liquid (Malamis et al., 2014), and biogas slurry (Chang et al., 2022; Chen et al., 2022), yielding significant results. In a study conducted by Chang et al. (2022) investigating the impact of the carbon-nitrogen ratio (C/N) on biogas slurry treatment within an activated sludge SCND system, it was observed that even with a reduced C/N ratio, NH_4_^+^-N and total nitrogen (TN) removal efficiencies could reach approximately 90%.


(3) $${NH}_{4}^{+}+1.5O_{2}\to 2H^{+}+NO_{2}^{-}+H_{2}O$$

(4) $$2NO_{2}^{-}+6H\mathrm{(electron donor)}\to 2OH^{-}+2H_{2}O+N_{2}$$

#### 2.1.2. Anaerobic ammonium oxidation

Anaerobic ammonium oxidation (ANAMMOX) technology involves the direct conversion of NH_4_^+^-N to N_2_ under stringent anaerobic conditions, facilitated by anaerobic ammonia-oxidizing bacteria (AnAOB) utilizing NO_2_^−^-N as the electron acceptor (Eq. 5; Kuenen, 2008). Therefore, ANAMMOX is well-suited for treating wastewater containing both NH_4_^+^-N and NO_2_^−^-N. Notably, electron acceptor NO_2_^−^-N can also be generated through short-cut nitrification (Eq. 3), making partial short-range nitrification an ideal precursor process for anaerobic ammonia oxidation (Wang J., 2021). The living conditions for AnAOB require strict anaerobic conditions, which contribute to energy savings by reducing aeration requirements. Moreover, the ANAMMOX process operates as an autotrophic process, eliminating the need for additional organic carbon sources, rendering it particularly suitable for denitrification in wastewater with low C/N ratios (Morales et al., 2015; Chini et al., 2019). Additionally, the stable pH levels maintained during ANAMMOX can preserve alkalinity, while AnAOB exhibits extended generation cycles and generates less sludge, substantially reducing the cost associated with excess sludge treatment and disposal (Jia et al., 2014; Ma et al., 2016).


$${NH}_{4}^{+}+1.3NO_{2}^{-}+0.13H^{+}+0.066HCO_{3}^{-}\to$$

(5) $$1.02N_{2}+0.066CH_{2}O_{0.5}N_{0.15}+2.03H_{2}O+0.26NO_{3}^{-}$$

#### 2.1.3. Simultaneous nitrification and denitrification

Simultaneous nitrification and denitrification (SND) technology involves the bio-denitrification process where nitrification and denitrification reactions take place simultaneously in both space and time. SND relies on the generation of an oxygen concentration gradient due to oxygen diffusion limitations, resulting in the formation of hypoxic microenvironments within the core of sludge flocs or within biofilms. These microenvironments support the coexistence of aerobic and hypoxic metabolic activities (Layer et al., 2020). This technology allows nitrification and denitrification reactions to occur in the same spatial domain, significantly reducing the required reactor volume (Third et al., 2005; Masoudi et al., 2018). Additionally, SND imposes minimal demands for aeration and organic carbon, resulting in a reduction of approximately 30% in sludge production compared to traditional bio-denitrification processes (Ma et al., 2017; Zhao et al., 2017). Furthermore, there is no need for sludge reflux, and the alkalinity consumed during the nitrification process can be replenished by the alkalinity generated during denitrification (Ling, 2020). This not only maintains reactor pH stability but also reduces costs and optimizes process operations.

The core of SND technology is the control of DO concentration, which can be categorized into aerobic, low oxygen, and ultra-low DO SND denitrification (Ling, 2020; Xiang, 2020). Temperature, pH, and the C/N ratio are also pivotal factors influencing SND technology (Ling, 2020). It’s worth noting that autotrophic bacteria have a considerably slower growth and reproduction rate in comparison to heterotrophic bacteria. In long-term operation, heterotrophic denitrifying bacteria tend to become the dominant microbial community, reducing the significance of autotrophic nitrifying bacteria and subsequently affecting the denitrification effectiveness of SND (Jia et al., 2020).

#### 2.1.4. Heterotrophic nitrification-aerobic denitrification

Robertson et al. (1985) achieved the successful isolation of a denitrifying Paracoccus bacterium, a heterotrophic microorganism with the unique ability to directly convert ammonia nitrogen in wastewater into gaseous nitrogen. These bacteria possess both heterotrophic aerobic nitrification and heterotrophic aerobic denitrification functions, enabling them to utilize organic matter to convert NH_4_^+^-N, NO_2_^¯^-N, and nitrate nitrogen (NO_3_^¯^-N) into either organic nitrogen or gaseous nitrogen. In recent years, it has become evident that such functional microorganisms are widespread in nature, including species like *Paracoccus pantotrophus*, *Acinetobacter* sp., *Phytophthora* sp., *Diaphorobacter* sp., *Alcaligenes faecalis*, among others (Robertson et al., 1985; Xian et al., 2016; Jia et al., 2019). This denitrification method is known as heterotrophic nitrification aerobic denitrification (HN-AD; Robertson and Kuenen, 1990).

The HN-AD process, akin to SND, offers structural integration, reduced footprint, and simplified operational management. Moreover, heterotrophic microorganisms exhibit higher growth rates and denitrification capabilities (Yang Y. et al., 2017; Zhang H. H. et al., 2019; Zhang M. Y. et al., 2019), bestowing HN-AD technology with the advantages of rapid start-up, stable operation, and resilience to oxygen and organic substrates. However, the reaction process requires a higher organic carbon source and aeration rate compared to traditional biological denitrification. Therefore, it is most suitable for denitrification and decarbonization treatment of wastewater with a high C/N ratio, albeit with increase energy consumption and operational costs (Chen et al., 2014; Huang et al., 2021).

#### 2.1.5. Granular sludge

Granular sludge is generally considered to be granular activated sludge that gradually forms a stable structure after condensing into flocs by free bacteria (Sarma et al., 2017). There are five theoretical mechanisms for its formation, including the filamentous bacteria hypothesis, selective pressure-driven hypothesis, extracellular polymer hypothesis, microbial self-aggregation hypothesis, and crystal nucleus hypothesis (Li, 2020). Depending on whether microorganisms require oxygen for growth, they can be divided into anaerobic granular sludge and aerobic granular sludge (AGS), with AGS being the more commonly used. Mature AGS typically exhibits an orange-yellow color, a smooth surface, and a spherical or ellipsoidal shape, with particle sizes ranging from 0.5 to 1.5 mm (Moy et al., 2002). The structure of AGS is dense and regular, with strong settling capabilities. Thanks to microorganisms’ inherent self-solidification properties, AGS maintains its structural integrity even under dynamic conditions, forming agglomerated organisms without swelling or negatively affecting water quality (Mota et al., 2014). This characteristic enables AGS to efficiently remove high concentrations of toxic organic substances, nitrogen, and phosphorus (Kishida et al., 2009; He et al., 2020; Chen H. Y. et al., 2023).

In practical applications, the key to this technology lies in the domestication and cultivation of AGS. Factors significantly affecting AGS formation and performance include fluid shear force, COD load, DO, settling time, particle size, temperature, hydraulic retention time, sludge age, and the presence or absence of induced nuclei (Pishgar et al., 2020).

#### 2.1.6. Other bio-denitrification technologies

In addition to the aforementioned technologies, novel bio-denitrification technologies also encompass sulfur autotrophic denitrification (Song et al., 2023), iron autotrophic denitrification (Zhao et al., 2022), hydrogen autotrophic denitrification (Dong et al., 2021), and other methods. While these autotrophic bio-denitrification technologies offer numerous advantages over heterotrophic bio-denitrification processes, such as conserving organic carbon sources, being suitable for treating low C/N ratio wastewater, and reducing sludge production, they do present some practical challenges in their application in wastewater treatment.

The research on sulfur autotrophic denitrification technology traces its origins back to the 1970s. Compared to other autotrophic denitrification methods, the reduced sulfides (S^0^, S^2−^, Na_2_S_2_O_3_) used as electron donors are cost-effective and readily available, less sensitive to water quality variations, and easy to utilize. Moreover, due to the inclusion of S oxidation and N reduction in the sulfur autotrophic denitrification process, there is significant potential for waste resource utilization (Kosgey et al., 2022). However, the sulfur autotrophic denitrification reaction generates H^+^, lowers the system’s pH, and produces a substantial amount of environmentally polluting sulfate that must be controlled. High concentrations of sulfides can also impact microbial activity, hinder autotrophic denitrification efficiency, and especially affect the conversion of nitrate to nitrite (Beristain et al., 2006). Some studies have combined sulfur autotrophic denitrification with microbial fuel cell technology to achieve simultaneous organic matter removal and electricity generation. However, the deposition of elemental sulfur can lead to electrode poisoning or scaling, posing challenges to the further advancement of this technology (Lin et al., 2018; Wang and He, 2020).

Iron autotrophic denitrification technology is an autotrophic bio-denitrification method that employs Fe^0^ or Fe^2+^ as electron donors (Zhao et al., 2022). Fe (II) is widely distributed in the environment and is cost-effective, but it presents challenges in maintaining system stability for continuous operation. Fe^0^, on the other hand, offers superior electron-donating potential compared to Fe (II), leading to a significant reduction in the redox potential of denitrification sludge and improved system stability (Zhang Y. H. et al., 2014; Yu Y. et al., 2022). Currently, nano zero-valent iron is a key area of research in the field of iron autotrophic denitrification (Mofradnia et al., 2019). Various factors influence iron autotrophic denitrification, including temperature, pH, and the Fe/N ratio (Johnson et al., 2007; Oshiki et al., 2013; Yu Y. et al., 2022). pH regulation is particularly impactful, although it can increase the operational complexity of the iron autotrophic denitrification process. Moreover, the understanding of the denitrification process mechanism in this technology remains somewhat limited, especially regarding the relationship between its biological and chemical aspects (Yu Y. et al., 2022). Additionally, the microbial population and metabolic pathways in the iron autotrophic denitrification process are not yet fully understood, and further exploration is required to comprehend the reaction mechanism between iron ions and other compounds.

Hydrogen autotrophic denitrification involves the use of hydrogen bacteria to denitrify and remove nitrogen using H_2_ as an electron donor (Dong et al., 2021). It boasts advantages such as high denitrification efficiency, rapid reaction rates, environmental cleanliness, and the ease of removing residual H_2_ from water without requiring additional treatment. However, there are safety concerns associated with the flammability and explosiveness of H_2_ when mixed with air, leading to transportation risks and elevated operation and maintenance costs. Additionally, the low solubility of H_2_ in water, with only 1.6 mg H_2_ dissolved per liter of water at 20°C, results in low utilization rates (30–50%). These factors have restricted the widespread adoption of this technology, and current research on hydrogen autotrophic denitrification remains largely in the laboratory stage (Zhang Y. B. et al., 2014).

### 2.2. Novel bio-denitrification coupling technology

In recent years, most researchers have coupled new biological denitrification technologies to maximize their individual advantages and compensate for the limitations of independent use. This has resulted in the development of economically and environmentally friendly bio-denitrification technologies that hold great promise. Examples include partial short-cut nitrification ANAMMOX (PN-ANAMMOX; Qiu et al., 2021; Wang Z., 2021; Shang et al., 2023; Yuan et al., 2023), ANAMMOX-SND (Yu Y. et al., 2022), sulfur autotrophic-iron autotrophic denitrification (Yang et al., 2023), iron autotrophic-hydrogen autotrophic denitrification (Liang et al., 2022), and bioelectrochemical denitrification (Huang et al., 2020a; Prarunchaya et al., 2021). In this context, the focus will primarily be on the short-range nitrification anaerobic ammonia oxidation process and the bioelectrochemical system.

#### 2.2.1. PN-ANAMMOX

PN-ANAMMOX is a novel coupled biological denitrification process discovered at a waste leachate treatment plant in the Mechernich region of Germany (Hippen et al., 1997). It combines the ANAMMOX process with the short-cut nitrification process, often uniting them within a single reactor (Lu et al., 2012; Wang Z., 2021). Short-cut nitrification entails the control of ammonia oxidation to the nitrite stage (Eq. 3), ensuring a continuous and stable supply of nitrite, the essential oxidation substrate for ANAMMOX. The ideal NO_2_^−^-N to NH_4_^+^-N ratio is recognized as 1.3 (Eq. 3), though practical applications typically achieve a ratio of approximately 1, reflecting the challenge of controlling the short-cut nitrification process (Wang Z., 2021). In comparison to traditional bio-denitrification, short-cut nitrification inherently reduces aeration energy consumption, while AnAOB necessitates strict hypoxia. Consequently, this process can save nearly 60% of the energy demand for aeration compared to traditional bio-denitrification (Zaborowska et al., 2018). The estimated energy consumption for traditional bio-denitrification stands at approximately 2.4 kW·h·kg^−1^, whereas the energy consumption of the PN-ANAMMOX process is approximately 1.0 kW·h·kg^−1^ (Figueroa et al., 2012; Liang et al., 2016). Furthermore, both short-cut nitrification and ANAMMOX are autotrophic processes, eliminating the need for additional organic carbon sources, making them highly suitable for denitrifying wastewater with low C/N ratios. Additionally, PN-ANAMMOX significantly reduces sludge production by around 90% (Morales et al., 2015; Zaborowska et al., 2018; Chini et al., 2019).

However, it’s important to note that the initiation and domestication of anaerobic ammonia-oxidizing bacteria are relatively time-consuming. AOB and NOB often coexist, and the removal of NOB from the system is one of the challenges of this process. Moreover, the denitrification efficiency of PN-ANAMMOX is generally influenced by process parameters such as DO concentration, COD concentration, heavy metals, temperature, pH value, and sludge age, contributing to the complexity of its operation (Lotti et al., 2012; Zuo et al., 2020).

#### 2.2.2. Bioelectrochemical systems

Bioelectrochemical systems (BESs) have emerged in recent years as a technology that combines microbiology with electrochemistry to achieve wastewater treatment and energy recovery (Liu and Logan, 2004). BESs rely on electrically active microbial catalytic electrodes, where oxidation reactions occur at the anode and reduction reactions happen at the cathode (Chang et al., 2016; Huang, 2021). Due to its sustainability (Yang W. L. et al., 2017), strong pollutant removal capability (Cao M. J. et al., 2020), and low sludge production (Wang and He, 2020), it has become a new type of low-energy-consumption water treatment technology that has attracted significant attention (Nguyen and Babel, 2022). Currently, many studies have used BESs to enhance various bio-reaction processes, including the removal of pollutants like nitrogen (Cecconet et al., 2020), phosphorus (Elmaadawy et al., 2020), organic matter (Cao et al., 2021), and heavy metals (Cao L. B. et al., 2020) that are challenging to degrade. Some studies have also employed BESs to improve denitrification in low C/N ratio wastewater treatment. The enhancement mechanisms involve the positive effects of bioelectricity on microbial functional enzymes and genes, as well as the direct provision of electrons by bioelectricity to the denitrification process, achieving electro autotrophic denitrification (Huang et al., 2020a, 2022). In addition, for denitrification in wastewater containing high concentrations of hard-to-degrade organic matter, BESs can break down these complex compounds into simpler organic matter, providing an organic carbon source for denitrification (Huang et al., 2020b). However, it’s important to note that BESs are primarily used for medium to low concentration nitrogen loads, and this technology is currently mainly under laboratory research, with the potential to become a mainstream nitrogen removal process in the future.

## 3. The denitrification performance

### 3.1. The denitrification performance of novel bio-denitrification technology

Section 2.1 introduces several emerging biological denitrification technologies while providing brief explanations of their application scope, advantages, disadvantages, and the factors influencing each technology. Among these aspects, the denitrification performance is a central concern for these innovative denitrification technologies. Table 1 presents the performance data related to nitrogen and carbon removal during the application of some of these novel biological denitrification technologies.

**Table 1:** The denitrification performance of a novel bio-denitrification technology.

Bio-denitrification technology	Denitrification system	Wastewater	Average nitrogen load	Influent C/N ratio	Average nitrogen and carbon removal efficiency			Ref
					COD	NH_4_^+^-N /NO_3_^¯^-N	TN	
SCND	Activated sludge	Landfill leachate	100 mg NH_4_^+^-N·L^−1^	4.0–8.0	89%	99%	82%	Zhang et al. (2020)
	Membrane aerated biofilm reactor	Synthetic wastewater	62 mg NH_4_^+^-N·L^−1^	4.0	97%	96%	72%	Chang et al. (2022)
	Moving-bed biofilm reactor	Synthetic wastewater	48 mg NH_4_^+^-N·L^−1^	3.6	-	-	81–88%	Francesca et al. (2021)
	Fixed biofilm-activated sludge (IFAS)	Biogas slurry	400–800 mg NH_4_^+^-N·L^−1^	11.7	-	94%	92%	Chen et al. (2022)
			600–800 mg NH_4_^+^-N·L^−1^	6.2	-	91%	86%	
ANAMMOX	Activated sludge	Pharmaceutical wastewater	100 mg NH_4_^+^-N·L^−1^	1.3	-	-	87%	Chen H. Y. et al. (2023)
	Expanded granular sludge bed	Synthetic inorganic wastewater	1,200 mg N·L^−1^	1.1	-	-	95%	Jiang et al. (2023)
SND	Fixed bed folded plate bioreactor	Mariculture wastewater	120 mg NH_4_^+^-N·L^−1^	4.0	-	99%	-	Guo et al. (2023)
	New air lift bioreactor	Urban sewage	40 mg NH_4_^+^-N·L^−1^	3.8	-	-	>90%	Li Y. et al. (2023)
	Composite sequencing batch biofilm reactor	High salinity wastewater	40 mg NH_4_^+^-N·L^−1^	10	96%	99%	91%	Li M. et al. (2023)
	Moving bed biological reactor	Synthetic wastewater	500 mg NH_4_^+^-N·L^−1^	1.28	-	85–90%	91%	Roumi and Debabrata (2023)
HN-AD	Activated sludge	Chemical wastewater and pig farming wastewater	200–1,600 mg NH_4_^+^-N·L^−1^	3, 5, 10, 15, 20, 25	-	>98%	-	Chen P. P. et al. (2023)
	Activated sludge	High salinity wastewater	94 mg NH_4_^+^-N·L^−1^	5–15	-	98%	-	Huang et al. (2023)
AGS	Dehydrated sludge particles as inoculant	Artificially synthesized urban sewage	11 mg NH_4_^+^-N·L^−1^	19	90%	95%	70%	Sun et al. (2023)
Other	Sulfur autotrophic denitrification filter	Secondary effluent from sewage treatment plant	12 mg NO_3_^¯^-N·L^−1^	1.8	-	95%	-	Song et al. (2023)
	sulfur autotrophic denitrification filter	Landfill leachate	125 mg NO_3_^¯^-N·L^−1^	0.1	-	-	90%	Zhang et al. (2023)
	Hydrogen autotrophic membrane bioreactor	Domestic wastewater	80 mg NO_3_^¯^-N·L^−1^	2	-	88%	-	Dong et al. (2021)
	FeS_2_ autotrophic denitrification filter	Synthetic wastewater	20 mg NO_3_^¯^-N·L^−1^	0.5	-	90%	-	Zhao et al. (2022)

As shown in Table 1, these novel bio-denitrification technologies have been employed in various wastewater treatment processes, including membrane bioreactors (Chang et al., 2022; Chen et al., 2022) and biofilters (Song et al., 2023). Remarkably, these technologies are not limited to synthetic wastewater treatment (Francesca et al., 2021; Chang et al., 2022; Zhao et al., 2022; Jiang et al., 2023; Roumi and Debabrata, 2023; Sun et al., 2023) but have also demonstrated their effectiveness in treating practical wastewater sources such as landfill leachate (Zhang et al., 2020, 2023), biogas slurry (Chen et al., 2022), urban domestic sewage (Dong et al., 2021; Li Y. et al., 2023), secondary effluent from sewage treatment plants (Song et al., 2023), aquaculture wastewater (Chen H. Y. et al., 2023; Guo et al., 2023), and chemical wastewater (Chen H. Y. et al., 2023; Chen P. P. et al., 2023). These novel bio-denitrification technologies exhibit versatility in handling a wide range of nitrogen concentrations, with initial nitrogen loads ranging from as high as 1,600 mgN·L^−1^ (Chen P. P. et al., 2023) to as low as 11 mgN·L^−1^ (Sun et al., 2023). Importantly, they consistently achieve robust denitrification effects, with denitrification efficiencies surpassing 70%, and in most cases, approaching 100%.

As shown in Table 1, autotrophic biological denitrification technology is primarily employed for the advanced treatment of low C/N ratio wastewater and has demonstrated significant advantages. Anaerobic ammonia oxidation technology, sulfur autotrophic, hydrogen autotrophic, and iron autotrophic biological denitrification technologies all exhibit treatment efficiencies exceeding 85% when applied to wastewater with a C/N ratio less than 3 (Dong et al., 2021; Zhao et al., 2022; Chen H. Y. et al., 2023; Jiang et al., 2023; Song et al., 2023; Zhang et al., 2023). Even in the study by Zhao et al. (2022), where iron autotrophic biological denitrification technology was employed to treat wastewater with an extremely low C/N ratio of 0.5, a denitrification efficiency of 90% was achieved. The widespread use of these autotrophic bio-denitrification technologies has the potential to effectively reduce chemical costs (related to organic carbon sources) and expenses associated with excess sludge treatment, aligning with the global low-carbon concept that has gained prominence in recent years.

### 3.2. The denitrification performance of novel bio-denitrification coupling process

Section 2.2 introduces several novel bio-denitrification coupling technologies, and Table 2 provides data on denitrification and carbon removal performance during the application of selected coupling denitrification technologies. As demonstrated in Table 2, the actual wastewater treated by these new biological denitrification coupling processes includes landfill leachate (Wang Z., 2021), domestic wastewater (Huang et al., 2020a; Prarunchaya et al., 2021; Qiu et al., 2021; Liang et al., 2022; Shang et al., 2023; Yang et al., 2023; Yuan et al., 2023), and chemical wastewater (Wang et al., 2022; Yu D. Y. et al., 2022; Jia et al., 2023), closely resembling the wastewater types addressed by the new biological denitrification technologies in Table 1. PN-ANAMMOX technology has emerged as the most prevalent coupling method in recent years, showcasing its versatility in handling a wide range of nitrogen loads and consistently achieving robust denitrification efficiency exceeding 90% (Qiu et al., 2021; Wang Z., 2021; Shang et al., 2023; Yuan et al., 2023).

**Table 2:** The denitrification performance of a novel bio-denitrification coupling technology.

Novel bio-denitrification coupling technology	Wastewater	Average nitrogen load	Influent C/N ratio	Average nitrogen and carbon removal efficiency			Ref
				COD	NH_4_^+^-N/ NO_3_^¯^-N	TN	
PN-ANAMMOX	Landfill leachate	1,454 mg NH_4_^+^-N·L^−1^	3.0	-	93%	-	Wang Z. (2021)
PN-ANAMMOX	Rural domestic sewage	121 mg NH_4_^+^-N·L^−1^	2.0	86%	-	90%	Yuan et al. (2023)
PN-ANAMMOX	Urban sewage	60 mg NH_4_^+^-N·L^−1^	2.8	>76%	-	92%	Qiu et al. (2021)
PN-ANAMMOX	Domestic sewage	50 mg NH_4_^+^-N·L^−1^	3.0	-	78%	-	Shang et al. (2023)
SND-ANAMMOX	Collagen sleeve wastewater	79–208 mg NH_4_^+^-N·L^−1^	1.0–1.8	-	94%	80%	Yu Y. et al. (2022)
Hydrogen autotrophic denitrification in bioelectrochemical systems	Synthetic wastewater	20 mg NO_3_^¯^-N·L^−1^	1.0	-	74%	54%	Prarunchaya et al. (2021)
SND in the multi-anode microbial fuel cells	Synthetic wastewater	64 mg NH_4_^+^-N·L^−1^	3.5	94%	-	71%	Huang et al. (2020a)
Heterotrophic nitrification aerobic denitrification coupled AGS	Synthetic petroleum wastewater	65 mg NH_4_^+^-N·L^−1^	1.5–12.3	-	92%	80%	Wang et al. (2022)
Enhanced iron and hydrogen autotrophic denitrification by biofilm coupled microelectrolysis of iron scrap	Synthetic wastewater	28 mg NH_4_^+^-N·L^−1^	19	91%	93%	80%	Liang et al. (2022)
Sulfur autotrophic coupled iron autotrophic denitrification system	Synthetic wastewater	50 mg NO_3_^¯^-N·L^−1^	0	-	-	90%	Yang et al. (2023)
Anaerobic nitrification coupled SND	Pharmaceutical wastewater	200 mg NH_4_^+^-N·L^−1^	52.5	97%	96%	85%	Jia et al. (2023)

The granular sludge technology primarily involves introducing granular sludge into the system, which, while shortening the reactor’s startup time, does not exhibit high adaptability or resistance to sewage impact (Sun et al., 2023). Consequently, researchers have explored its combination with other bio-denitrification technologies to cultivate specific granular sludge for wastewater treatment (Wang et al., 2022). However, the TN removal efficiency of this process has proven to be less than ideal. For instance, in a study by Sun et al. (2023), isolated AGS was applied to treat artificially synthesized urban wastewater, achieving a 95% removal efficiency for ammonia nitrogen, but only a 70% removal efficiency for TN. Similarly, Wang et al. (2022) utilized “heterotrophic nitrification aerobic denitrification” in conjunction with AGS to treat synthetic petroleum wastewater, attaining a 92% removal efficiency for ammonia nitrogen, while the TN removal efficiency reached only 80%. Therefore, whether used independently or in conjunction with other technologies, its TN removal efficiency falls short of that for ammonia nitrogen. Further investigation is needed to better understand the underlying reasons and explore potential enhancement measures.

Transportation safety risks associated with H_2_ limit the application of hydrogen autotrophic denitrification (Zhang Y. H. et al., 2014). However, BES with external electric fields can produce hydrogen *in situ*, providing a local source of H_2_ for hydrogen autotrophic denitrification (Prarunchaya et al., 2021). Nevertheless, the extra electricity needed for this coupled technology increases its operational costs, to some extent, restricting its large-scale practical application. Additionally, sulfur autotrophic denitrification faces challenges such as low pH values and secondary pollution due to sulfate by-products. The sulfur autotrophic coupled with iron autotrophic denitrification system effectively reduces sulfate by-products and exhibits robust denitrification performance (Yang et al., 2023).

## 4. Application prospect

### 4.1. Upgrading and renovation of urban sewage treatment plants

The novel bio-denitrification technology represents a promising sewage treatment approach with broad engineering applications. It effectively addresses the challenges associated with traditional denitrification processes, such as lengthy processing, substantial land requirements, high energy consumption, and significant financial investments (Zhang et al., 2022). In comparison to conventional biological denitrification methods, this innovative technology offers superior efficiency and adaptability. It can cater to the treatment requirements of various wastewater types and achieve efficient deep denitrification in low C/N ratio wastewater without the need for additional external carbon sources. Therefore, in light of current domestic and international policies aimed at improving effluent standards in urban sewage treatment plants, the novel bio-denitrification technology emerges as a favorable low-carbon choice.

### 4.2. Coupling with other technologies

The novel bio-denitrification technology not only compensates for the limitations of independent use through self-coupling but also pairs effectively with non-biological methods to provide reference parameters that are challenging to regulate in practical engineering. For instance, in treating highly concentrated toxic and hazardous wastewater, physical and chemical techniques such as adsorption, membrane separation, electrochemistry, and oxidation–reduction can be employed as pretreatment. Subsequently, they can be combined with biological denitrification technology for advanced treatment, thereby further expanding the practical application range and improving sewage treatment efficacy. The greater challenge for the future is to explore low-energy, cost-effective technologies while maximizing process coupling to overcome constraints in real-world wastewater scenarios and enable large-scale applications. This development direction aligns with the low-carbon concept and introduces innovative ideas for environmentally friendly denitrification.

### 4.3. The reduction of greenhouse gases emission

Chan-Pacheco et al. (2021) explored the substantial potential of combining new bio-denitrification technologies for the purification and reduction of denitrification gas pollutants, including N_2_O, CH_4_, and H_2_S, which are presently significant greenhouse gases of global concern. This suggests that future research could delve into additional technology coupling approaches for greenhouse gas emission reduction, further contributing to the low-carbon concept.

### 4.4. Smart water management

In the practical application of novel bio-denitrification technologies, the cultivation and regulation of microbial communities are key factors for achieving efficient denitrification. However, challenges can arise, such as unstable environmental conditions or increased operating costs due to the need for adjustments in temperature, pH, and other parameters when cultivating these microorganisms. Therefore, it is worth considering the utilization of novel bio-denitrification technologies within the realm of smart water management, where the integration of automation, computer technology, and other advancements can standardize the domestication and cultivation of microorganisms. Simultaneously, this approach can help reduce the reliance on human resources and propel the advancement of new biological denitrification technology towards broader practical implementation.

## 5. Conclusion

This paper reviews the denitrification performance of novel bio-denitrification technologies and their combined applications, analyzes the recent research situation, and summarizes the future development direction and challenges of these technologies. Novel bio-denitrification technology is environmentally friendly and holds vast application prospects, aligning with the sustainable low-carbon development concept. With ongoing technological progress, novel bio-denitrification technologies are expected to expand their presence in various fields, contributing significantly to environmental protection and sustainable development.

## Author contributions

SH: Funding acquisition, Investigation, Writing – original draft, Writing – review & editing. YLF: Investigation, Writing – original draft. HMZ: Writing – review & editing. CQW: Writing – review & editing. CLZ: Writing – review & editing. XGL: Writing – review & editing.
